# Coping Strategies and Their Relationship With Subjective Distress due to the COVID-19 Pandemic in Brazil

**DOI:** 10.1177/00332941221110538

**Published:** 2022-06-29

**Authors:** Juliana Alvares Duarte Bonini Campos, Lucas Arrais Campos, Bianca Gonzalez Martins, João Marôco

**Affiliations:** School of Pharmaceutical Sciences, São Paulo State University (UNESP), São Paulo, Brazil; Faculty of Medicine and Health Technology, 7840 Tampere University, Tampere, Finland; 28108 Tampere University Hospital, Tampere, Finland; School of Dentistry, Campus Araraquara, São Paulo State University (UNESP), São Paulo, Brazil; School of Pharmaceutical Sciences, São Paulo State University (UNESP), São Paulo, Brazil; William James Center for Research (WJCR), University Institute of Psychological, Social, and Life Sciences (ISPA), Lisbon, Portugal

**Keywords:** coping strategies, pandemic, COVID-19, subjective distress

## Abstract

**Objectives:**

To identify the strategies used by Brazilian adults for coping with the COVID-19 pandemic and to verify the effect of these strategies on subjective distress.

**Methods:**

This was a cross-sectional observational study with online data collection in May/June 2020, November/December 2020, and May/June 2021. The BriefCOPE Inventory and the Impact of Event Scale (IES-R) were used. The prevalence of strategies used at different time points was estimated with a 95% confidence interval and compared with a z-test. A multiple logistic regression model was constructed and the odds ratio (OR, 95%CI) was calculated to verify the probability of subjective distress according to the coping strategy used.

**Results:**

Younger individuals had a lower prevalence of adaptive strategies, which increased significantly with age. Participants with higher income levels had a higher prevalence of adaptive strategies, as did those who were never diagnosed with a mental health disorder. The prevalence of using only maladaptive strategies ranged from 6.1% to 5.4% (*p* > 0.05). The use of problem-centered strategies (Active Coping and Planning), venting of emotions, and substance use increased with time, while acceptance and behavioral disengagement decreased. In general, the population used problem-centered strategies, but the high prevalence of problem avoidance was striking. Positive reinterpretation and acceptance were protective factors for subjective distress, whereas maladaptive strategies increased the chance of distress. The presence of a negative valence component (problem- or emotion-centered) increased the chance of subjective distress, whereas strategies based on Problem Solving acted as a protective factor.

**Conclusion:**

Coping strategies were significantly associated to subjective distress and have changed since the beginning of the pandemic. Strategies focused on emotion regulation may be relevant to minimize distress.

## Introduction

Since the beginning of the COVID -19 pandemic, the world population has been coping with the health crisis and its consequences. The virus, the lack of control, the unpredictability, and the need to adopt behaviors to prevent virus infection and disease transmission (e.g., use of masks, hand sanitation, isolation, and social distancing) have changed daily routines and lifestyles and require additional cognitive and behavioral efforts to cope with the new context of life.

Identifying the strategies that individuals use to cope with a problem, in this case the COVID -19 pandemic, and its consequences, may therefore be important for understanding the process of adaptation, recovery, and development in the face of adversity (Lazarus & Folkman, 1984). Carver (1997) reports that the coping strategies used may be predictors of future psychological outcomes, which include *Subjective Distress* and *Post-traumatic Stress*, underscoring the need for their study.

In the theoretical framework proposed by Lazarus and Folkman (1984), coping is a dynamic process that changes with time and phase, with the stressful situation, as well as with the presence or absence of emotional, social, and material resources of the individual. In this perspective, the prolongation and unpredictable end of the pandemic must be considered, as the mobilization of resources for coping can be adjusted as needed. We must also consider the Brazilian context, which has chronic and severe economic, educational, and social inequalities. These inequalities have intensified during the pandemic and may represent an external source of demands (Campos et al., 2020; Serafim et al., 2021) that, in addition to the internal demands, may exceed the individual’s coping skills and hinder the use of healthy, flexible, and well-adapted coping strategies.

A series of studies (Agha, 2021; Bhattacharjee & Acharya, 2020; Ciulkowicz et al., 2021; Fukase et al., 2021; Gori et al., 2020; Gurvich et al., 2021; Kolodziejczyk et al., 2021; Serafim et al., 2021) were conducted online showing the coping strategies used by people from different samples and countries since the beginning of the COVID-19 pandemic. Serafim et al. (2021) examined the coping strategies of 3000 Brazilian adults (women: 83.0%, mean age = 39.8 years) during May-June 2020 and found that 61.3% of participants reported increased hours of sleep and 40.8% reported increased intake of food, alcohol, drugs, tobacco, and medications. The authors suggest that these behaviors indicate the adoption of the negative strategy of avoidance, attempting to mask the problem rather than act on its solution, which is a risk behavior for the development of health-related symptoms. Fukase et al. (2021) conducted a 1-week study of 2708 Japanese adults (women: 50.0%; mean age = 49.2 years) in July 2020 and found that adopting the coping strategies of Planning (PL) and Instrumental Social Support (IS) were protective factors for the development of depression, whereas Behavioral disengagement (BD) and Self-blame (SB) were significant risk factors.

Ciulkowicz et al. (2021) identified different psychopathological responses resulting from the use of various coping strategies during the COVID-19 pandemic (March and April 2020) among Polish medical (*n* = 1173) and non-medical (*n* = 658) workers. Three groups of coping strategies were identified. Group 1 (G1), labeled Non-specific, included participants who scored below average on all subscales of the BriefCOPE Inventory. Group G2, labeled Maladaptive, included participants who scored above average on the Denial (DN), Substance Use (SU), BD, SB, and Venting of Emotions (VE) subscales. Group 3 (G3) included individuals with above-average scores on the Active Coping (AC), PL, Positive Reinterpretation (PR), Emotional Social Support (ES), and IS. The G2 participants had more somatic symptoms, anxiety, insomnia, social dysfunction, major depression, and subjective distress than G1 and G3. When comparing between the two worker groups in G1 and G3, no significant differences in mental stress scores were found. Also in Poland, Kolodziejczyk et al. (2021) determined the psychological impact of the COVID-19 pandemic and the coping strategies of 2036 participants (79.0% women; mean age=39.4 years) in the period from March to April 2020. A positive and significant association was found between the use of maladaptive coping strategies (DN, SU, BD, AC, VE) and psychopathological symptoms and distress. A positive and significant relationship was also found between subjective distress and the adoption of Religion (RE) as a coping strategy. Adaptive strategies (AC, PL, Acceptance – AT, Humor – HU) were negatively correlated with psychopathological symptoms.

Agha (2021) examined the relationship between coping strategies and mental health in 100 professionals (83 men and 17 women, mean age = 33.13 years) at a university institution in Saudi Arabia who were under lockdown for 6 weeks due to COVID-19. Coping strategies related to maladaptive (SB, BD, SU, VE, and Self-distraction – SD) were observed more frequently in men than in women, and posed an increased risk for the development of depression, anxiety, and stress. Problem-focused strategies (AC, PL, IS, ES) were not significantly associated with psychological symptoms. Gurvich et al. (2021) studied 1495 Australian adults (women: 81.6%, mean age = 42.5 years) from April to May 2020 and found that SD, BD, and SB were associated with higher depression scores. In addition, BD, SB, and IS were associated with higher anxiety scores, whereas scores were lower in people with AT. In the study by Gori et al. (2020), 1102 Italian adults (females: 70.0%, mean age = 34.91 years) participated and problem-focused coping strategies were found to act as mediators between life satisfaction and perceived stress.

So far, no studies have assessed coping strategies used at different times during the COVID-19 pandemic in a given population. Moreover, the literature on mental health in the pandemic has grown exponentially, but there is still little information on coping strategies. Such studies can provide useful evidence for the development of interventions that can help people understand their abilities and difficulties in dealing with a life problem. These studies can also help identify and promote healthy ways of coping with negative events, allowing for the establishment of appropriate individual contexts that can minimize the impact of the pandemic on the mental health of the population.

There are several theoretical models and measurement tools for assessing coping strategies in the literature. Stanislawski (2019) presents a general summary of these concepts, models, and instruments, which helps researchers and clinicians in the selection of a model. The most commonly used instruments include the Ways of Coping Questionnaire (WCQ (Lazarus & Folkman, 1984)), the Multidimensional Coping Inventory (MCI (Endler & parker, 1990)), the Coping Inventory for Stressful Situations (CISS (Endler & Parker, 1999)), and the COPE Inventory (Carver et al., 1989). These instruments assess coping responses that focus on the problem and also address situational aspects, identifying responses as potentially dysfunctional or adaptive and problem-centered or emotion-centered (Carver, 1997). However, the grouping of coping strategies (coping styles) in different samples and contexts is inconsistent in the literature (Baumstarck et al., 2017; Carver et al., 1989; Cooper et al., 2006; Daruy-Filho et al., 2013; Hastings et al., 2005). The COPE inventory is a widely used instrument that originally had 60 items distributed among 15 factors. After its use in different samples and contexts, redundancies were found in the content of the items, and a reduced version of the instrument (BriefCOPE) consisting of 28 items distributed over 14 factors was developed (Carver, 1997). Of the original 15 factors, 2 were excluded and a new factor (SB) was added.

Lazarus (1966) and Compas (1987) state that coping strategies depend on the stressor, the context, and the characteristics and culture of the sample and that the effectiveness of the coping measures are variable and flexible. For these reasons, the assessment and grouping of coping strategies, as well as the interpretation of outcomes, should be specific for the population and context under study. The coping style, i.e., a combination of strategies that are preferred to perform a particular function and that are relatively stable, is thus defined (Carver et al., 1989). Given that coping is not a homogeneous concept and that the extraction of higher-order factors may not represent the ideal theoretical model for the interpretation of coping styles, Stanislawski (2019) proposes the use of the Coping Circumplex Model (CCM) as a basis for assessment. This model relies on a bipolar dimension of coping, with a problem-centered and emotion-centered coping types, representing a circular continuum of coping styles. The CCM includes four bipolar dimensions composed of eight coping styles; positive emotional coping, efficiency, problem solving, preoccupation with the problem, negative emotional coping, helplessness, problem avoidance, and hedonic disengagement. The authors (Stanislawski, 2019) propose that the strategies evaluated with different measurement tools (WCQ, CISS, COPE) can be integrated in the CCM coping styles, and therefore, the model may add to the findings and considerations about the prevailing coping style in different samples and contexts.

In view of the above, this study was developed with the aim of determining the coping strategies of Brazilian adults in dealing with the COVID-19 pandemic at three different time points and to examine the contribution of these strategies to the occurrence of subjective distress.

## Methods

### Study Design and Sampling

This was a cross-sectional observational study conducted among the adult population (≥18 years of age) with data collection at three time points during the COVID-19 pandemic in Brazil (Stage 1: May to June 2020, Stage 2: November to December 2020, and Stage 3: May to June 2021^1^). Data were collected online via a link to Google Forms (Stages 1 and 2) and/or to Lime Survey^2^ (Stage 3).

The minimum sample size was calculated based on the estimate provided by the World Health Organization that mental health disorders account for 12% of the global burden of diseases (World Health Organization, 2001). We used α = 5%, sampling error=10%, and *N* = 167762351 (Brazilian population ≥15 years of age^3^, estimated for 2020 at https://www.ibge.gov.br/). The minimum estimated sample size for each survey stage was 2818 people (229 from the North, 750 from the Northeast, 1208 from the Southeast, 412 from the South, and 219 from the Midwest).

To characterize the sample, information such as sex, age (years), monthly family income (in Brazilian reals - R$), and self-reported medical diagnosis of a mental health disorder before the COVID-19 pandemic was collected. Age was categorized based on previous studies (Campos et al., 2020, 2021) into (<24, 24├33, 33├43, 43├55 and ≥55 years). The BriefCOPE inventory was used to assess coping strategies used during the pandemic (Carver, 1997; Maroco et al., 2014) and subjective distress was estimated using the Impact of Event Scale-Revised (IES-R) (Caiuby et al., 2012).

### Measuring Instruments

The BriefCOPE Inventory is a self-administered instrument consisting of 28 items grouped into 14 scales (Active Coping – AC, Planning – PL, Instrumental Support – IS, Emotional Support – ES, Religion – RE, Positive Reinterpretation – PR, Self-Blame – SB, Acceptance – AT, Venting of emotions – VE, Denial – DN, Self-Distraction – SD, Behavioral Disengagement – BD, Substance Use – SU, and Humor – HU) (Carver, 1997). The Portuguese version used in this study was adapted from Maroco et al. (2014) to the Portuguese spoken in Brazil and Portugal. This version has a 5-point Likert-type response scale (0: I’ve never done this; 1: I’ve done this before; 2: Sometimes; 3: I usually do this; and 4: I always do this).

To assess participants' subjective distress, we used the revised IES-R proposed by Weiss and Marmar (1997). The IES-R consists of 22 items distributed across three factors (avoidance, intrusion, and hyperarousal) that are added to an overall psychological distress score. The instrument has a 5-point Likert-type response scale ranging from 0 to 4 (0 – not at all, 1 – slightly, 2 – moderately, 3 – very, and 4 – extremely). In this study, the Portuguese version proposed by Caiuby et al. (2012) was used.

### Procedures and Ethical Aspects

The survey link was sent to participants via email, WhatsApp, or social media and remained open for response for 40 days. The non-probability snowball method was used for data collection. Initial contacts were made via email with professors at various higher education institutions in Brazil that had email addresses available on the websites. They were asked to forward the link to the survey to their contacts, thus expanding the reach of the study. Instructions were also provided for disseminating the link via email, WhatsApp, or social networks. This study was approved by the National Research Ethics Committee of the Ministry of Health (CONEP) (CAAE 30,604,220.4.0000.0008).

### Data Validity and Reliability

Data validity was estimated using confirmatory factor analysis (CFA) with robust weighted least squares method adjusted for mean and variance (WLSMV). The fit of the BriefCOPE and IES-R models to the data was assessed using the Comparative Fit Index (CFI), Tucker-Lewis Index (TLI), and Root Mean Square Error of Approximation (RMSEA) indices. The fit was considered adequate when CFI and TLI≥0.90 and RMSEA≤0.10 (Kline, 1998). The factor loading (λ) of the items were evaluated and considered satisfactory when λ ≥ 0.50. Reliability was analyzed with the ordinal alpha coefficient (α), and α ≥ 0.70 was considered adequate (Table 1).

**Table 1.** Psychometric indicators related to fit of the models (BriefCOPE Inventory and Impact of Event Scale - revised (IES-R)) to the samples.

			CFA^a^				
Measuring Instrument	Stage^a^	n	λ	CFI	TLI	RMSEA [90%CI]	α^†^
BriefCOPE^b^	1	12921	0.68–0.98	0.991	0.987	0.049[0.048–0.050]	0.71–0.97
	2	6162	0.68–0.98	0.989	0.984	0.056[0.055–0.057]	0.73–0.96
	3	7922	0.73–0.98	0.990	0.985	0.052[0.051–0.053]	0.71–0.96
Invariance (CFI)	M0:Configural = 0.990; M1:Metric = 0.990; M2:Scalar = 0.990 ჻ ΔCFI = 0 (M1-M0; M2-M1)						
IES-R^c^	1	12921	0.51–0.89	0.965	0.960	0.072[0.071–0.073]	0.84–0.92
	2	5927	0.55–0.88	0.974	0.971	0.064[0.062–0.065]	0.85–0.91
	3	7894	0.53–0.88	0.972	0.968	0.067[0.066–0.069]	0.82–0.94
Invariance (CFI)	M0:Configural = 0.968; M1:Metric = 0.968; M2:Scalar = 0.973 ჻ ΔCFI < 0.01 (M1-M0; M2-M1)						

^a^CFA: confirmatory factor analysis with robust weighted least squares method adjusted for mean and variance (WLSMV), Comparative Fit Index (CFI), Tucker-Lewis Index (TLI), Root Mean Square Error of Approximation (RMSEA) estimated with a 90% confidence interval [90%CI]; †α: ordinal alpha coefficient; ##Stage 1: May to June 2020, Stage 2: November to December 2020, Stage 3: May to June 2021.

^b^BriefCOPE: refined model excluding factor Humor.

^c^IES-R: refined model excluding item 2.

The fit of the BriefCOPE and IES-R to the samples was adequate, demonstrating the validity and reliability of the data. To compare the results obtained at different stages of data collection, we tested the metric and scalar invariance of the instruments between samples (Cheung & Rensvold, 2002) using the CFI difference (ΔCFI) and reductions <0.01 indicated model invariance. Models were invariant across samples, allowing comparison of coping strategies and psychological effects between samples (Table 1). The MPLUS v.8.3 program (Muthén and Muthén, Los Angeles, CA) was used to conduct the analyses.

### Statistical Analysis

After fitting the model to the data, two strategies were considered for the grouping of coping strategies. First, three theoretical factors (problem-centered adaptive coping (PCA), emotion-centered adaptive coping (ECA), and maladaptive coping (MA) (Carver et al., 1989)) were retained from the exploratory factor analysis. We used parallel analysis (KMO > 0.70; Bartlett's test: *p* < 0.001), the diagonally weighted least squares (DWLS) estimation method, and robust prominence rotation (Promin rotation) (Lorenzo-Seva & Ferrando, 2006; Timmerman & Lorenzo-Seva, 2011). The H-index was used (ACP = 0.954; ACE = 0.937; DES = 0.928) and the stability of the model was considered adequate (H-index > 0.80) (Ferrando & Lorenzo-Seva, 2018). This model was used at all stages of data collection. Factor retention was performed using the data from stage 1 and the following coping groups were established: PCA: Active Coping (AC), Planning (PL), Religion (RE), Positive Reinterpretation (PR), Acceptance (AT); ECA: Instrumental Support (IS), Emotional Support (ES), and Venting of Emotions (VE); MA: Self-Blame (SB), Denial (DN), Self-Distraction (SD), Behavioral Disinvestment (BD), and Substance Use (SU). This configuration was not considered a second-order hierarchical model, but merely groupings for theoretical and general framing of the BriefCOPE factors for the study sample. The program FACTOR (Lorenzo-Seva & Ferrando, Tarragona, Spain) was used to perform the analyses.

The mean value of each BriefCOPE factor was calculated and categorized into commonly used strategies (mean values ≥3) or rarely used strategies (<3). The prevalence of commonly used strategies was estimated with a confidence interval (95% CI) for each stage of data collection and for sex, monthly household income, age group, and previous diagnosis of a mental health disorder separately. The z test was used for comparison between stages or variables of interest.

For a second analysis strategy we used the CCM proposed by Stanislawski (2019) First each coping style was classified as problem- (P) or emotion (E)-focused coping with positive or negative valence. Then, coping styles were divided into problem solving (P+), problem avoidance (P-), positive emotional coping (E+), negative emotional coping (E-), efficiency (P+E+), helplessness (P-E-), preoccupation with the problem (P+E), and hedonic disengagement (P-E+). The equivalence was made between the CCM factors and each factor of the BriefCOPE presented by Carver et al. (Carver et al., 1989; Carver, 1997) (E+ = HU, RE; P+E+ = PR; P+ = AC, PL; E- = VE, SB; P-E- = DN; P- = SD; P-E+ = SU; P+E- =BD). The mean values of each coping style (CCM) were calculated. Means were then categorized into commonly used coping styles (means ≥3) or rarely used coping styles (<3), and the prevalence of commonly used strategies was estimated using 95% CI; the z test was used for comparison between stages.

To test the probability of moderate/severe psychological distress (y = mean IES-R score > 1.5; 1 = moderate/severe distress) as a function of the coping strategy used (Model 1: x_1_ to x_14_ = BriefCOPE factors, Model 2: x_1_ to x_8_ = CCM coping styles, reference category = 0 scores < 3), logistic regression was conducted and the odds ratio (OR]95%CI[) was calculated for each stage of data collection.

### Similarity Analysis

For this analysis, individuals who did not commonly use any of the BriefCOPE strategies (mean≥3) were excluded and the strategy most commonly used by the remaining individuals was identified. Because individuals can use one or more strategies in combination, we performed a similarity analysis based on graph theory to determine the relationships between the strategies commonly used by participants at each stage of the study. With this analysis, the frequency and relationship between the coping strategies used can be verified. Results were presented using a Fruchterman Reingold static graph generated with the program *Interface de R pour les Analyses Multidimensionnelles de Textes et de Questionnaires* - Iramuteq® version 0.7 alpha 2 (Ratinaud, Déjean and Skalinder, Laboratoire LERASS, Université Tolouse, France, 2008–2014).

## Results

Table 2 lists the characteristics of the samples in the three stages of data collection and the prevalence of participants using only adaptive strategies (PCA and/or ECA). The majority of participants were women, people under 55 years of age, and people from higher economic level. The prevalence of people with a pre-pandemic diagnosis of a mental health disorder was high (25.7–31.3%). A lower prevalence of adaptive strategies was found among younger people, and this prevalence increased significantly with age. The higher the participants' monthly income (R$), the higher the prevalence of exclusive use of adaptive strategies. Participants who had been diagnosed with a mental health disorder at some point in their lives prior to the pandemic had a significantly lower prevalence of exclusive use of adaptive strategies than participants without a medical diagnosis.

**Table 2.** Sample characteristics and prevalence (p, 95%CI) of participants from each time point who only and commonly (mean≥3) used adaptive strategies (PCA, ECA) to cope with the pandemic by sex, age group, income, and previous diagnosis of mental health disorder.

	^a^Stage 1 (*n* = 12921)				Stage 2 (*n* = 6162)				Stage 3 (*n* = 7922)			
Characteristic	n(%)	p	95%CI	p-value	n(%)	p	95%CI	p-value	n(%)	p	95%CI	p-value
Sex												
Men	3791 (29.4)	41.7	40.2–43.3	<0.001	1995 (32.4)	43.4	41.2–45.5	0.196	2533 (32.0)	44.7	42.7–46.6	0.087
Women	9.063 (70.1)	36.8	35.8–37.8		4135 (67.1)	41.6	40.1–43.1		5323 (67.2)	42.6	41.3–44.0	
Non-binary	-				2 (0.05)				42 (0.5)			
Other	-				3 (0.05)				12 (0.15)			
No response	67 (0.5)				27 (0.4)				12 (0.15)			
Age group (years)												
<24 (1)	2765 (21.7)	25.0	23.3–26.6	^1x2^<0.001	863 (14.6)	24.2	21.4–27.1	^1x2^<0.001	1325 (16.7)	27.1	24.7–29.5	^1x2^<0.001
24├33 (2)	3438 (27.0)	32.5	31.0–34.1	^2x3^<0.001	1472 (24.9)	34.0	31.6–36.5	^2x3^<0.001	1738 (22.0)	35.4	33.1–37.6	^2x3^0.001
33├43 (3)	3064 (24.0)	42.5	40.7–44.2	^3x4^<0.001	1495 (25.3)	44.6	42.1–47.1	^3x4^<0.001	1943 (24.5)	43.8	41.6–46.0	^3x4^<0.001
43├55 (4)	2107 (16.5)	51.6	49.5–53.8	^4x5^0.948	1203 (20.4)	53.0	50.2–55.9	^4x5^0.021	1700 (21.5)	52.8	50.5–55.2	^4x5^0.031
≥55 (5)	1378 (10.8)	51.5	48.9–54.2		874 (14.8)	58.1	54.9–61.4		1210 (15.3)	56.9	54.1–59.7	
Monthly family income (Brazilian reals – R$)												
0 to 1254 (1)	1117 (8.7)	27.9	25.3–30.6	^1x2^0.589	251 (4.1)	29.5	23.8–35.1	^1x2^0.926	363 (4.6)	32.8	28.0–37.6	^1x2^0.907
1255 to 2004 (2)	1502 (11.7)	28.9	26.6–31.2	^2x3^<0.001	511 (8.4)	29.2	25.2–33.1	^2x3^<0.001	620 (7.8)	32.4	28.7–36.1	^2x3^<0.001
2005 to 8640 (3)	4960 (38.6)	35.7	34.3–37.0	^3x4^<0.001	2350 (38.5)	37.4	35.4–39.3	^3x4^<0.001	2975 (37.6)	40.0	38.3–41.8	^3x4^<0.001
8641 to 11,261 (4)	2066 (16.1)	43.1	41.0–45.3	^4x5^0.003	1163 (19.0)	45.1	42.2–47.9	^4x5^0.001	1451 (18.4)	46.2	43.6–48.7	^4x5^0.076
≥11,262 (5)	3198 (24.9)	47.3	45.6–49.1		1830 (30.0)	51.5	49.2–53.8		2503 (31.6)	49.1	47.1–51.1	
Diagnosis of MHD^b^ before the pandemics												
No	8876 (68.7)	42.0	41.0–43.0	<0.001	4229 (70.2)	45.4	43.9–46.9	<0.001	5927 (74.3)	45.9	44.6–47.1	<0.001
Yes	4045 (31.3)	30.2	28.8–31.6		1794 (29.8)	33.1	30.9–35.3		2046 (25.7)	35.1	33.0–37.1	

^a^Stage 1: May to June 2020, Stage 2: November to December 2020, Stage 3: May to June 2021.

^b^MHD: mental health disorder.

Note. Estimated prevalence from Model 1, obtained by exploratory factor analysis for the data. PCA: problem-centered adaptive coping – Active Coping, Planning, Religion, Positive Reinterpretation, and Acceptance; ECA: emotion-centered adaptive coping – Instrumental Support, Emotional Support, and Venting of Emotions.

Few participants reported not using any of the strategies of the BriefCOPE (total score = 0: 14 people in stage 1, 4 in stage 2, and 16 in stage 3), and approximately 10% (stage 1: 11.8%, stage 2: 12.6%, and stage 3: 10.7%) of participants had mean scores of all factors < 3, indicating that the coping strategies were not regularly used. The prevalence of individuals using only maladaptive strategies (SB, DN, DC, BD, SD) was 6.1, 5.8, and 5.4% in stages 1, 2, and 3, respectively (z-test; *p* = 0.06–0.26).

Table 3 shows the prevalence of commonly used (mean≥3) coping strategies of the BriefCOPE in the three stages of data collection. In the third stage of data collection, a significant increase in the use of active coping and planning strategies was observed. Acceptance and behavioral disengagement decreased and venting of emotions and substance use increased with time. The highest use of religion as a coping strategy was at the beginning of the pandemic (stage 1).

**Table 3.** Prevalence (p, 95%CI) of commonly used (mean≥3) coping strategies and mean scores of the different coping strategies of the BriefCOPE at different stages of data collection.

	Stage 1		Stage 2		Stage 3	
BriefCOPE	p	95%CI	p	95%CI	p	95%CI
Active coping (AC)	37.4^b^	36.6–38.3	35.6^a^	34.4–36.8	43.9^c^	42.8–45.0
Planning (PL)	45.4^a^	44.5–46.3	47.2^b^	45.9–48.4	51.6^c^	50.5–52.7
Positive reinterpretation (PR)	31.8^b^	31.0–32.6	31.2^a,b^	30.1–32.4	30.0^a^	29.0–31.0
Acceptance (AT)	38.7^b^	37.9–39.6	37.7^b^	36.5–38.9	35.0^a^	33.9–36.0
Religion (RE)	37.2^c^	36.4–38.0	30.6^a^	29.4–31.7	34.7^b^	33.6–35.7
Instrumental support (IS)	20.6^a^	19.9–21.3	24.1^b^	23.0–25.1	23.0^b^	22.1–24.0
Emotional support (ES)	32.6^a^	31.8–33.4	32.5^a^	31.3–33.7	32.9^a^	31.9–34.0
Venting of emotions (VE)	23.0^a^	22.3–23.8	23.9^a^	22.8–24.9	25.4^b^	24.5–26.4
Self distraction (SD)	38.5^c^	37.7–39.3	31.4^a^	30.2–32.5	35.1^b^	34.0–36.1
Substance use (SU)	5.6^a^	5.2–6.0	5.5^a,b^	5.0–6.1	6.3^b^	5.8–6.8
Denial (DN)	2.2^a^	2.0–2.5	2.1^a^	1.7–2.4	2.3^a^	2.0–2.6
Self-blame (SB)	12.7^a^	12.1–13.3	17.8^b^	16.8–18.8	12.9^a^	12.2–13.7
Behavioral disengagement (BD)	7.5^b^	7.1–8.0	6.2^a^	5.6–6.9	6.5^a^	6.0–7.1
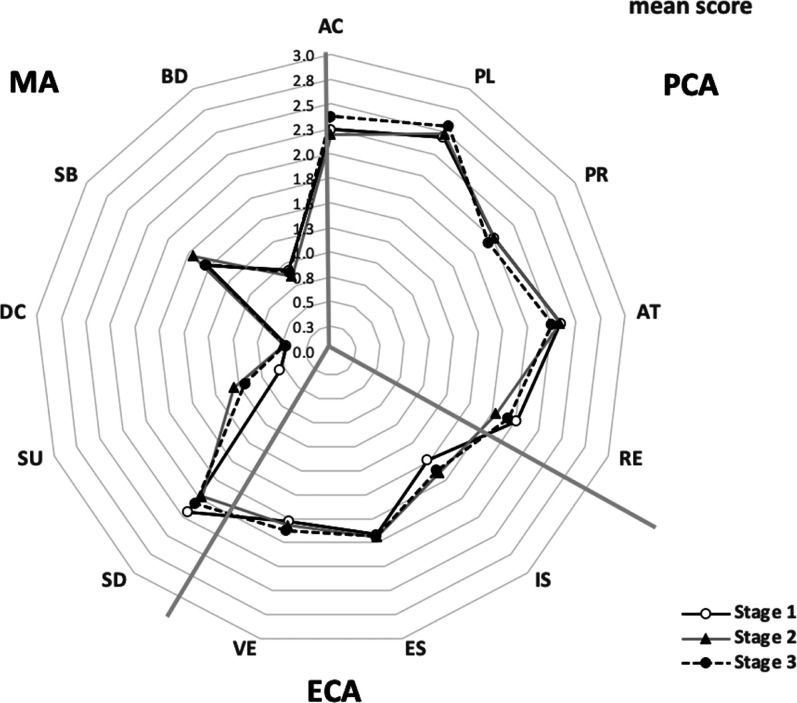						

Note. Humor factor was excluded (see Confirmatory Factor Analysis). Different lowercase letters indicate statistical difference between stages (z test, α = 0.05). PCA: problem-centered adaptive coping; ECA: emotion-centered adaptive coping; MA: maladaptive coping.

Table 4 shows the mean scores of coping styles represented in the CCM and the prevalence of individuals with a mean score ≥3 in each of these styles at different time points of data collection. In general, the population was focused on problem solving (facing the pandemic). Although the proportion of individuals using problem avoidance decreased from the first to the other stages of data collection, the high prevalence of this coping style is outstanding, suggesting that some people avoided thinking about and dealing with the problem. In the second time point, there was a significant increase in emotionally negative strategies.

**Table 4.** Mean scores of coping styles represented in the Coping Circumplex Model (CCM) and prevalence (p, 95%CI) of individuals with a mean score ≥3 in each of the styles at different stages of data collection.

Coping Style (CCM)		Stage 1		Stage 2		Stage 3	
		p	95%CI	p	95%CI	p	95%CI
Problem solving	P+	34.2^a^	33.4–35.0	33.9^a^	32.7–35.1	40.6^b^	39.5–41.7
Efficiency	P+E+	31.8^b^	31.0–32.6	31.2^a,b^	30.0–32.4	30.0^a^	29.0–31.1
Positive emotional coping	E+	5.7^a^	5.3–6.1	6.8^b^	6.2–7.4	6.0^a,b^	5.5–6.5
Hedonic disengagement	P-E+	5.6^a^	5.2–6.0	5.5^a^	4.9–6.1	6.3^a^	5.8–6.8
Problem avoidance	P-	38.5^c^	37.7–39.3	31.4^a^	30.2–32.6	35.1^b^	34.0–36.1
Helplessness	P-E-	2.2^a^	1.9–2.4	2.1^a^	1.7–2.5	2.3^a^	2.0–2.6
Negative emotional coping	E-	8.7^a^	8.2–9.2	10.6^b^	9.8–11.4	8.7^a^	8.1–9.3
Preoccupation with the problem	P+E-	7.5^b^	7.0–7.9	6.2^a^	5.6–6.8	6.5^a^	6.0–7.0
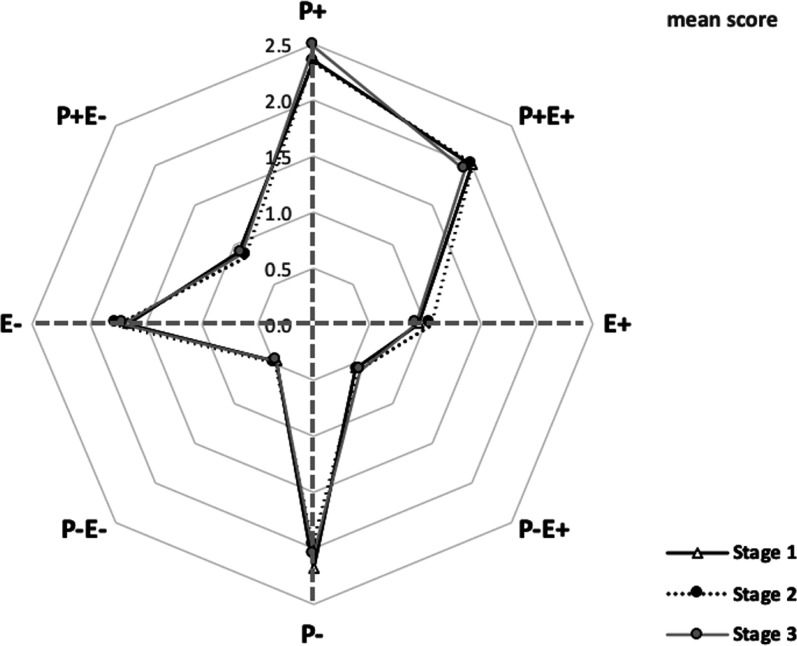							

Note. Different lowercase letters indicate statistical difference between stages (z test, α = 0.05).

Regarding subjective distress, about one-third of the sample (stage 1: 35.2%, stage 2: 30.5%, and stage 3: 37.0%) reported being moderately or severely affected by the pandemic, with a significant decrease in stage 2 but an increase in stage 3 (z-test, *p* < 0.001). Table 5 shows the likelihood of experiencing subjective distress due to the pandemic according to the coping strategies most commonly used by participants, considering the strategies from the BriefCOPE and the CCM.

**Table 5.** Odds ratio (OR, 95% CI) of subjective distress (IES-R mean score ≥1.5) as a function of coping strategies used by participants at each stage of data collection.

		Stage 1			Stage 2			Stage 3		
		OR	95%CI	p-value	OR	95%CI	p-value	OR	95%CI	p-value
Model^a^										
PCA	Active coping (AC)	0.77	0.69–0.86	<0.001	0.86	0.73–1.01	0.067	0.76	0.66–0.86	<0.001
	Planning (PL)	0.80	0.72–0.89	<0.001	0.87	0.75–1.02	0.082	0.93	0.82–1.06	0.288
	Positive reinterpretation (PR)	0.48	0.43–0.53	<0.001	0.61	0.51–0.71	<0.001	0.47	0.41–0.54	<0.001
	Acceptance (AT)	0.61	0.56–0.67	<0.001	0.66	0.57–0.75	<0.001	0.61	0.54–0.69	<0.001
	Religion (RE)	1.04	0.95–1.14	0.359	0.91	0.79–1.06	0.226	1.09	0.97–1.23	0.132
ECA	Instrumental support (IS)	1.23	1.10–1.39	<0.001	1.18	1.00–1.40	0.050	1.27	1.10–1.46	0.001
	Emotional support (ES)	1.38	1.25–1.53	<0.001	1.26	1.08–1.47	0.003	1.24	1.09–1.41	0.001
	Venting of emotions (VE)	2.01	1.82–2.21	<0.001	2.03	1.76–2.34	<0.001	1.85	1.64–2.09	<0.001
MA	Self distraction (SD)	2.29	2.10–2.50	<0.001	2.33	2.04–2.66	<0.001	2.33	2.09–2.61	<0.001
	Substance use (SU)	2.83	2.35–3.41	<0.001	3.143	2.42–4.08	<0.001	3.51	2.79–4.42	<0.001
	Denial (DN)	2.98	2.17–4.09	<0.001	3.29	2.03–5.33	<0.001	4.88	3.16–7.56	<0.001
	Self-blame (SB)	3.56	3.14–4.04	<0.001	3.27	2.81–3.81	<0.001	3.77	3.20–4.44	<0.001
	Behavioral disengagement (BD)	3.18	2.69–3.76	<0.001	2.58	2.00–3.32	<0.001	3.71	2.90–4.74	<0.001
2 – CCM^c^										
E+	Positive emotional coping	0.96	0.80–1.14	0.631	0.91	0.71–1.17	0.470	1.12	0.90–1.39	0.299
P+E+	Efficiency	0.44	0.40–0.48	<0.001	0.53	0.46–0.62	<0.001	0.43	0.38–0.49	<0.001
P+	Problem solving	0.69	0.63–0.76	<0.001	0.79	0.69–0.92	0.002	0.79	0.70–0.88	<0.001
E-	Negative emotional coping	5.29	4.54–6.16	<0.001	4.38	3.62–5.29	<0.001	3.54	2.93–4.28	<0.001
P-E-	Helplessness	3.71	2.71–5.10	<0.001	4.28	2.65–6.93	<0.001	5.50	3.58–8.46	<0.001
P-	Problem avoidance	2.34	2.15–2.54	<0.001	2.40	2.11–2.73	<0.001	2.32	2.09–2.58	<0.001
P-E+	Hedonic disengagement	3.10	2.58–3.72	<0.001	3.63	2.82–4.68	<0.001	3.73	2.98–4.67	<0.001
P+E-	Preoccupation with problem	3.57	3.03–4.20	<0.001	3.05	2.38–3.91	<0.001	4.46	3.52–5.65	<0.001

^a^Model 1: the 14 factors of BriefCope were considered, the factors retained by exploratory factor analysis (PCA: problem-centered adaptive coping, ECA: emotion-centered adaptive coping, MA: maladaptive coping) were presented for interpretation purposes only.

^b^Humor factor was excluded (see Confirmatory Factor Analysis).

^c^Model 2: Coping Circumplex Model – CCM.

**Note.** Multiple logistic model (y=moderate/severe subjective distress; IES-R≥33; Model 1: x_1_ a x_13_: BriefCOPE factors; Model 2: coping styles CCM – rc: reference category=0, rarely used coping styles, mean score< 3.

Model 1: Stage 1: -1.054-0.259_AC_-0.218_PL_+0.211_IS_+0.323_ES_-0.738_PR_-0.488_AT_ +0.828_SD_+0.696_VE_+0.043_RE_+1.270_SB_+1.093_DN_+1.158_BD_+1.041_SU._

Stage 2: -1.399-0,151_AC_-0.137_PL_+0.169_IS_+0.230_ES_-0.503_PR_-0.422_AT_ +0.846_SD_+0.708_VE_-0.090_RE_+1.184_SB_+1.190_DN_+0.947_BD_+1.145_SU._

Stage 3: -1.003-0,278_AC_-0.071_PL_+0.240_IS_+0.216_ES_-0.755_PR_-0.498_AT_ +0.848_SD_+0.614_VE_+0.091_RE_+1.326_SB_+1.586_DN_+1.310_BD_+1.256_SU._

Model 2: Stage 1: -0.935-0.044_E+_-0.824_P+E+_-0.371_P+_+1.665_E-_+1.311_P-E-_+0.848_P-_ +1.130_P-E+_+1.272_P+E-._

Stage 2: -1.231-0.091_E+_-0.635_P+E+_-0.230_P+_+1.477_E-_+1.455_P-E-_+0.876_P-_ +1.290_P-E+_+1.115_P+E-._

Stage 3: -0.841+0.115_E+_-0.831_P+E+_-0.241_P+_+1.264_E-_+1.705_P-E-_+0.842_P-_ +1.316_P-E+_+1.495_P+E-._

Positive reinterpretation and acceptance coping strategies were protective factors for subjective distress. The use of maladaptive strategies (SB, DN, BD, SD, and SU) significantly increased the likelihood of subjective distress (risk factor) in the context of the pandemic. Venting of emotions and instrumental and emotional support were also factors associated with subjective distress. The CCM showed that the presence of a negative valence component (problem- or emotion-centered) significantly increased the likelihood of moderate/severe subjective distress, whereas problem-solving strategies (P+) acted as a protective factor. Positive emotions showed no significant association with subjective distress.

**Figure 1.**
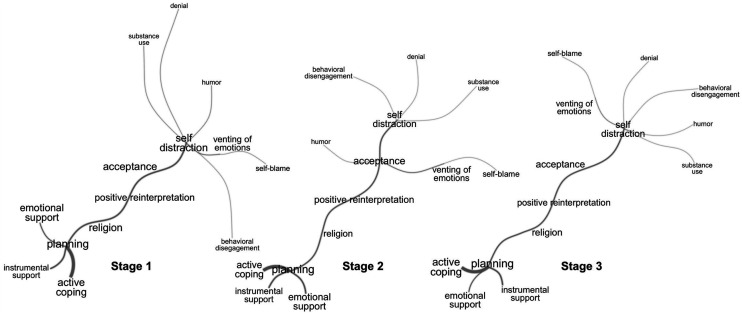
Similarity analysis of coping strategies used by participants in each stage of data collection (mean score≥3).

The similarity graphs in Figure 1 show that in stages 1 and 3 there were two main coping strategies (planning and self-distraction) that connected with different strategies used. The “Planning” strategy had an important connection to “Active Coping” and Social Support (emotional, instrumental), while the “Self-Distraction” strategy connected with five other strategies that could be ways of avoiding the problem. In stage 2, on the other hand, there were three main strategies: “Planning”, “Self-distraction,” and “Acceptance”. The planning strategy connected similarly to that of the other stages. The three connections of the “Self-distraction” strategy were maladaptive strategies, while the “Acceptance” core divided into two: Self-blame and Humor. Interestingly, self-blame was related to venting of emotions in all three stages.

## Discussion

This study examined for the first time the strategies used by the adult Brazilian population to cope with the COVID-19 pandemic and their relationship with subjective distress, at three time points. The validity and reliability of the obtained data supported the quality of the results. Our results may be useful for planning and developing support and care strategies aimed at preventing and minimizing the psychological damage of the pandemic and maintaining or restoring the well-being and mental health of the population. In addition, the data was analyzed using both the theoretical framework presented by Carver et al. (Carver et al., 1989; Carver, 1997) and the CCM (Stanislawski, 2019) to broaden the perspective for evaluating the coping strategies used. The use of two models may help clinicians interpret the coping mode (set of coping strategies) used by individuals to guide intervention planning, increasing the clinical applicability of the findings presented here.

Problem-solving strategies were a protective factor for subjective distress in the sample, and the use of such strategies increased from the first to the third stage of data collection, suggesting that a proportion of the population (∼33%) expanded its repertoire of coping skills over time. Perhaps, a better understanding of the pandemic occurred with time, which allowed for more efficient planning and action to eliminate or decrease the daily effects of the pandemic, thus causing a cognitive restructuring that led to greater psychosocial adaptation (Lazarus & Folkman, 1984). Nevertheless, a reasonable number of individuals (∼33%) were reluctant to think about the pandemic (P-), reflecting individual characteristics such as feelings of uncertainty, powerlessness, or lack of control over the pandemic (Rettie & Daniels, 2021). This could also reflect the rise of negationist ideologies, misinformation and false information disseminated in the social media that has occurred in Brazil (Beer, 2021; Jesus, 2020; Modesto et al., 2020). The phenomenon has become a risk, as it is related to the neglect of preventive measures for COVID-19, such as social distancing, use of masks and hand hygiene, increasing the likelihood of exposure to Sars-Cov-2.

The cores and connections identified in the similarity analysis indicate the coping style of the Brazilian population. Coping style is the set of coping strategies used to respond to a particular set of circumstances (dispositional factors). The findings confirm the political polarization of the Brazilian population that is reflected in the views and actions towards the pandemic, already highlighted by Modesto (Modesto et al., 2020). Two main coping styles were found in the population, which can be categorized according to Miller's proposal (1981) into monitoring (information seeking under threat: PL, AC, IS, ES) and blunting (information avoidance: SD, VE, SB, DN, BD, HU, SU). The person with a blunting coping style moves away from the threat, gets distracted, and avoids information by postponing an action. The person with monitoring coping style, on the other hand, is attentive and alert, seeking information and support to try to control the situation (Antoniazzi et al., 1998; MIller, 1981). Our results show that the latter may protect against the development of subjective distress, indicating a better adaptation of the individual to the pandemic context. Adopting the blunting style, on the other hand, significantly increases the likelihood of distress, which may contribute to a further decline in well-being and mental health, as reported in previous studies (Budimir et al., 2021; Campos et al., 2020, 2021).

The appearance of "Acceptance" as a node in the graph at the second time point (1 year after the start of the pandemic) can be attributed to people recognizing the seriousness of the pandemic after the initial shock and lack of information in the first wave of COVID-19. After 6 months (stage 3), the initial coping styles reappear. The relationship between venting of emotions and self-blame in the three stages of the study can be explained by the existing association between venting of emotions and rumination, which in turn is related to anxiety, hypervigilance, intrusive thoughts, self-criticism, and self-blame (Stanislawski, 2019). Thus, we can speculate that, when facing the problem (pandemic) and its consequences in daily life, the individuals who vent their feelings can also have negative and persistent thoughts, are more self-critical, and feel somewhat responsible for events directly or indirectly related to the pandemic.

We found that emotion-centered strategies significantly increased the likelihood of distress. Although emotions can occur naturally when faced with daily demands, controlling their valence and magnitude may be important in reducing the impact of a stressor on well-being and quality of life (Budimir et al., 2021; Leahy et al., 2011; Panayiotou et al., 2021; Restubog et al., 2020) especially during crises. Emotional control involves mental and behavioral processes that affect the onset, maintenance, and modification of reactions to experiences (Gross, 2014) and can be addressed with various psychotherapeutic techniques (Leahy et al., 2011). Psychoeducation and psychological support for emotional control strategies, such as expressive writing, emotional communication with others (social support), and encouragement of psychological flexibility, may be effective in mitigating the negative effects of crisis situations (Budimir et al., 2021; Panayiotou et al., 2021). Having a vast repertoire of emotional control strategies and the ability to use them with flexibility has been shown to have a positive impact on mental and physical health and quality of life. Therefore, actions towards a greater emotional understanding and an building an expanded set of coping strategies should be encouraged to minimize distress (Brehl et al., 2021; Leahy et al., 2011).

The lower frequency of exclusive use of adaptive coping strategies among younger individuals may be due to the smaller cognitive and behavioral repertoire for coping with the pandemic in this age group than in older individuals. Cheng et al. (2014) also suggest that older people generally have a greater ability to adapt to life demands because of the greater number of experiences and life patterns they accumulated over time and because of the adaptations required for a healthy aging process. This process leads to greater flexibility in adjusting expectations and, consequently, better psychological adjustment. The higher prevalence of exclusively adaptive coping strategies among people of higher economic status may, in turn, be related to this population's better access to health care, education, healthy food, and preventive measures and greater possibility of quarantine and social isolation. This context implies less exposure to additional stressors from the pandemic, allowing for better assessment of the situation and the selection of more effective coping strategies.

Participants with a previous a mental disorder also had a lower prevalence of using only adaptive coping strategies. A study by Bridi et al. (2018) found that patients with bipolar disorder used adaptive strategies significantly less than control patients. The authors attributed this to possible functional and emotional impairment, a more tense family environment, and greater emotional overload. In addition, long-term mental illness may lead to less effective mechanisms to deal with stressful situations. Thus, people previously diagnosed with a mental disorder are more vulnerable in the pandemic, and the lower use of adaptive strategies increases the likelihood of psychological symptoms and distress (Campos et al., 2021), which deserves attention.

This study has limitations, such as the non-probability sample design, which does not allow generalization of the results to populations with different characteristics, and the online data collection, which limited study participation to individuals with internet access and higher levels of education. However, given the pandemic scenario and social isolation measures, this was the feasible method for data collecting. Another limitation of our results that should be considered is that the medical diagnoses of mental health disorders were obtained by self-reported and had no clinical verification. However, the item used asked specifically about being medically diagnosed with a mental health disorder, and for the exploratory aim of this study, this information was considered sufficient.

Despite these limitations, we hope that the results presented here can help to underscore the need to plan population care strategies that focus not only on meeting the psychological needs that may arise from the pandemic, but also guide people to identify their beliefs, values, and feelings to increase the possibility of adopting more appropriate coping strategies that will lead to the maintenance of the population's physical and mental health and well-being. We suggest that future studies should include other psychological variables, such as perceived stress, perceived control, social support and personality traits, in order to verify their relationship with coping strategies and subjective distress.

## Conclusion

The type of coping strategy used by the respondents had a significant relationship with subjective distress. In general, Brazilians used strategies that were either protective or risk factors for distress. Emotional control strategies may minimize distress, and demographic characteristics should be considered in the development of actions aimed at increasing the use of healthy means to cope with the pandemic and creating more adapted individual contexts.

## Notes

1 Number of new cases of COVID-19: Stage 1–6/25/2020, last day of survey, *n *= 51147 with a 7-day moving average of 45665; Stage 2–12/25/2020, last day of survey *n *= 24615, moving average = 40797; Stage 3–6/25/2021, last day of survey *n *= 79277, moving average = 74471; deaths/day for COVID -19: Stage 1 - *n *= 1141; moving average = 1032; Stage 2 - *n *= 506, moving average = 691; Stage 3 - *n *= 2001, moving average = 1806 https://github.com/CSSEGISandData/COVID-19. Vaccination against COVID-19 began in Brazil on 17/01/2021. By 6/25/2021, 30.4% of the population had received the first dose of the vaccine and 10.9% had received the two doses (https://qsprod.saude.gov.br/extensions/DEMAS_C19Vacina/DEMAS_C19Vacina.html).

2 The data collection platform was changed because the new Guidelines for Research Procedures in Virtual Environment (CONEP) http://conselho.saude.gov.br/images/Oficio_Circular_2_24fev2021.pdf were published on February 24, 2021. Also, the Limesurvey platform had more tools available than that of Google Forms and allow for easier customization of data collection. The change had no direct impact on data collection.

3 The age of ≥15 years as the cutoff value was used because of the age groups established by IBGE. As we included only individuals aged ≥18 years, the estimated sample size would be greater than actually needed and thus meet the requirements of the study.
